# Supplementary material for: Probiotics Regulating Inflammation via NLRP3 Inflammasome Modulation: A Potential Therapeutic Approach for COVID-19
Source: Microorganisms. 2021 Nov 17;9(11):2376. doi: 10.3390/microorganisms9112376 (PMC8624812; doi:10.3390/microorganisms9112376)
Supplement: Supplementary file 1 [file microorganisms-09-02376-s001.zip › microorganisms-1427789-supplementary.pdf]

SUPPLEMENTAL MATERIAL

Author, Year of publication	Subjects	Sample Size	Experimental Protocol/Case of study	Group	Intervention	Treatment	Duration	Samples	NLRP3 Activation
Schmitz S. et al., 2015(41)	Canine	12 *in vivo*	Canine chronic enteropathy	CONTROL (Healthy)	Standardised hydrolyzed protein diet	*Enterococcus faecium* 1 x 10^{9} CFUs or placebo (maltodextrin)	6 weeks	Duodenal and colonic biopsies	Not significant difference between groups
				STUDY(Chronic Enteropathy)					
		28 dogs *ex vivo* (*)		CONTROL (Healthy)	*Ex-vivo* challenge (priming) with PBS, *Enterococcus faecium*, Flagellin, Pam_3CSK_4, LPS	PBS / *Enterococcus faecium* 1 x 10^{7} CFUs / *Flagellin* / Pam_3CSK_4	5 hours	Macrophag e DH82 cells	Minor effects on mRNA levels of the NLRP3 components
				STUDY (Chronic Enteropathy)		PBS / *Enterococcus faecium* 1 x 10^{7} CFUs / *Flagellin* / Pam_3CSK_4 / LPS			
Teixeira et al., 2018(32)	Rats	92	Gut microbiota contribution in type 1 diabetes development	CONTROL	Diabetes Prone-Rats	PBS	4 weeks	Ileal tissue	*L. johnsonii* N6.2 suppresses NLRP3 inflammasome
				STUDY 1		Rosmarinic acid			
				STUDY 2		*L. johnsonii* N6.2 1×10^{8} CFU			
				STUDY 3		Rosmarinic acid & *L. johnsonii* N6.2			
Li et al., 2018(29)	Porcine	48	Intestinal barrier function	CONTROL	Oral challenge with ETEC 1×10^{9} CFU/kg or saline	Basal Diet	2 weeks	Jejunal mucosa	NLRP3 attenuation with *Clostridium butyricum* pretreatment
				STUDY		*Clostridium butyricum* 3×10^{8} CFU/day -supplemented diet			
Chung et al., 2019(36)	Mice	28	Colitis-Associated Colorectal Cancer	CONTROL 1	Azoxymethane intraperitoneal (i.p) + DSS (in drinking water)	Wild type + PBS	5 weeks (34 days)	Colon tissue	Pretreatment with heat-killed *Enterococcus. faecalis* attenuates NLRP3 inflammasome activation in macrophages
				CONTROL 2		NLRP3 Knockout + PBS			
				STUDY 1		Wild type + heat-killed *Enterococcus faecalis* strain KH2 (17 mg/kg) p.o./day			
				STUDY 2		NLRP3 Knockout + heat-killed *Enterococcus faecalis* (17 mg/kg) p.o./day			
	Rats			CONTROL	HFD	Standard diet			

Luo et al., 2019(37)		72 (36 mothers and 36 offsprings)	HFD-induced hepatic oxidative stress, lipid disorders, and NLRP3 inflammasome activation in mother rats and offspring	STUDY 1		HFD	5 weeks (38 days)	Plasma and liver samples	Microbe-derived antioxidant supplementation during pregnancy and lactation attenuated HFD-induced NLRP3 inflammasome activation
				STUDY 2		HFD with 2% microbe-derived antioxidant (Fermented by *Bacillus subtilis, Lactobacillus*, Beer yeast)			
Ding et al.,201(4)9	Rats	60	Sepsis induced liver injury and hypoxic hepatitis after cecal ligation and puncture	CONTROL 1	Sepsis induction via cecal ligation and puncture	Sham + Sterile Water	72 hours	Serum and liver samples	LGG treatment decreased NRLP3 levels and reduced liver injury
				CONTROL 2		Sepsis + Sterile Water			
				STUDY 1		Sham + *Lactobacillus rhamnosus GG* (LGG)			
				STUDY 2		Sepsis + *Lactobacillus rhamnosus GG* (LGG)			
Wu et al., 2016(39)	Bovine	35 x 10^5 cells	*E. coli*-induced mastitis	CONTROL 1	*Ex-vivo* challenge with *E. coli*	Untreated BMECs	48 hours	BMECs	Attenuation of NLRP3 inflammasome activation
				CONTROL 2		*E. coli* (3 x 10^7 CFUs)			
				STUDY 1		*Lactobacillus rhamnosus* GR-1 (LRGR) (3 x 10^7 CFUs)			
				STUDY 2		*E. coli* (3 x 10^7 CFUs) + *L. rhamnosus* GR-1 (LRGR) 3 x 10^7 CFUs) pretreatment			
Avolio et al., 2019(30)	Syrian Hamsters	42	Unpredictable chronic mild stress followed by HFD	CONTROL 1	HFD & UCMS	Unstressed + normal diet + probiotic mix	4 weeks	Blood and brain	Decrease in expression levels of NLRP3 in hypothalamus, along with the circulating levels in blood
				CONTROL 2		Unstressed + HFD (without probiotics)			
				CONTROL 3		UCMS + normal diet (without probiotics)			
				CONTROL 4		UCMS + HFD (without probiotics)			

				STUDY 1		Unstressed + HFD +probiotic mix			
				STUDY 2		UCMS + normal diet + probiotic mix			
				STUDY 3		UCMS + HFD + probiotic mix			
Tohno et al., 2011(38)	Porcine	2 × 10^6 cells	Adult and newborn GALT	CONTROL	*Ex-vivo* challenge (priming) with MDP, iE-DAP, Pam_3CSK_4, and CpG	None	6 hours	Spleen, esophagus, duodenum, jejunum, ileum, colon tissues, Pps and MLNs	In the newborn the expression of NLRP3 was higher in the spleen and MLNs and lower in the other intestinal tissues. In the adult the expression of NLRP3 in the lower intestinal tissues were the same with the spleen. Increased expression in GALT (ileal Pps & MLNs). Two *Lactobacilli* strains enhanced NLRP3 expression in the GALT of adult and newborn swine.
				STUDY 1		MDP			
				STUDY 2		iE-DAP			
				STUDY 3		Pam_3CSK_4			
				STUDY 4		CpG			
				STUDY 5		L. *delbrueckii subsp. bulgaricus* NIAI B6			
				STUDY 6		*L. gasseri* JCM1131			
Bai et al., 2020(43)	Mice	20	Obesity	CONTROL	Bacteriocins produced by *L. helveticus*: PJ4, *L. brevis:* DT24 and *L. animalis*: TSU4	Normal diet	200 days	Feces, blood, adipose tissue	Increased expression of NLRP3 in all groups-especially in HFD- in comparison with controls. *L. brevis* DT24 did not result in any changes, while *L. helveticus* PJ4 + *L. animalis* TSU4 were able to reverse efficiently the deleterious effects of HFD.
				STUDY 1		HFD			
				STUDY 2		HFD + *L. brevis* DT24 (H+D)			
				STUDY 3		HFD + *L. helveticus* PJ4 (H +P)			
				STUDY 4		HFD + *L. animalis* TSU4 (H+T)			
	Rats	40		CONTROL	Formula	Formula	7 days		

Fan et al., 2019(34)			*Cronobacter sakazakii*-Induced NEC in neonatal rats	STUDY 1	Oral challenge with the *C. sakazakii* strain (1 x 10^9 CFU)	*Bacteroides fragilis* ZY-312 (1 x 10^9 CFU)		Feces, blood, and intestinal tissues	ZY-312 suppress *C. sakazakii*-induced NEC by modulating NLRP3 expression
				STUDY 2		Formula			
				STUDY 3		*Bacteroides fragilis* ZY-312 (1 x 10^9 CFU)			
Wu et al., 2020(31)	Mice	18	DSS-induced Colitis	CONTROL	Water	150 µl normal saline i.p. once daily	7 days	Colon and spleen tissues	Decreased expression of NLRP3
				STUDY 1	DSS in drinking water	150 µl normal saline i.p. once daily			
				STUDY 2		50 mg/kg *Roseburia intestinalis*-derived flagellin (R.I flagellin) in 150 µl normal saline i.p. once daily			
Xia et al., 2020(33)	Porcine	18	*Salmonella infantis*-Induced Enteritis	CONTROL	Sterile normal saline	Sterile normal saline	18 days	Jejunal and ileal tissues	*L. johnsonii* L531 inhibits the activation of NLRP3
				STUDY 1	Oral challenge with *S. Infantis* (1 × 10^{11} CFU/ ml, 10 ml/piglet)	Sterile normal saline *L. johnsonii* L531 (1 × 10^9 CFU/ml, 10 ml/day) given intragastrically			
				STUDY 2					
Kern et al., 2017(42)	Porcine	8 sows+16 piglets *In vivo*	ETEC	CONTROL	Oral challenge with ETEC	Untreated	Piglets: 29 days Sows: >29 days (long-term) *	Jejunal, ileal and colonic tissues	In 29-day-old piglets, the expression of NLRP3 was significantly higher compared with that in the ileum of 70-day-old pigs. Probiotic supplementation had no significant effect on mRNA expression of NLRP3.
				STUDY 1		*Enterococcus faecium* NCIMB 10415 *(E. faecium)* 4 × 10^6 CFU			
		5 piglets Ex vivo		CONTROL	---------	---------	150–180 minutes	Porcine intestinal	Expression of NLRP3 was higher in
				STUDY 1		*E. faecium* NCIMB 10415			

				STUDY 2	Incubation with ETEC $4x10^9$per well	---------		epithelial cells	epithelia mono-incubated with *E. faecium* or ETEC compared with epithelia incubated with *E. faecium* and ETEC
				STUDY 3		*E. faecium* NCIMB 10415			
Loss et al., 2018(40)	Porcine	8×10^5 cells *Ex vivo*	ETEC	CONTROL	*Ex-vivo* Challenge (priming) with LPS and incubation with ETEC 3×10^4 per well	Water	1.5, 6 and 20 hours	MoDC	ETEC stimulates a time-dependent inflammasome response. *E. faecium* did not stimulate NLRP3.
				STUDY 1		LPS			
				STUDY 2		LPS+ *E. faecium* NCIMB 10415			
				STUDY 3		*LPS+ETEC*			
				STUDY 4		*LPS+ETEC+ E. faecium* NCIMB 10415			
				CONTROL	Incubation with ETEC 3×10^4 per well	Water			
				STUDY 1		*E. faecium* NCIMB 10415			
				STUDY 2		ETEC			
				STUDY 3		*ETEC+ E. faecium* NCIMB 10415			

Not defined; ETEC: enterotoxigenic *Escherichia coli;* Pam$_3$CSK$_4$: Pam3CysSerLys4; LPS: lipopolysaccharide; PBS: phosphate buffered saline; GALT: gut associated lymphoid tissue; MDP: muramyldipeptide; iE-DAP: γ-d-glutamyl-*meso*-diaminopimelic acid; CpG: *CpG* sites; DSS: dextran sulfate sodium; MoDC: monocyte-derived dendritic cells; Pps: Peyer's patches; MLNs: mesenteric lymph nodes; HFD: high fat diet; NEC: Necrotizing Enterocolitis; UCMS: unpredictable chronic mild stress; BMECs: bovine mammary epithelial cells; CFUs: colony forming units.
